# The Effects of Gamma-Aminobuytric Acid (GABA) Enrichment on Nutritional, Physical, Shelf-Life, and Sensorial Properties of Dark Chocolate

**DOI:** 10.3390/foods12010213

**Published:** 2023-01-03

**Authors:** Wee Yin Koh, Xiao Xian Lim, Eva Sheue Wen Teoh, Rovina Kobun, Babak Rasti

**Affiliations:** 1 Faculty of Food Science and Nutrition, Universiti Malaysia Sabah, Kota Kinabalu 88400, Sabah, Malaysia; 2 Food Technology Division, School of Industrial Technology, Universiti Sains Malaysia, Minden 11800, Pulau Pinang, Malaysia; 3 Australasian Nanoscience and Nanotechnology Initiative, 8054 Monash University LPO, Clayton, VIC 3168, Australia

**Keywords:** amino acid, functional food, chocolate, gamma-aminobutyric acid, enrichment

## Abstract

Hypertension is the leading cause of cardiovascular disease and premature death worldwide. Gamma-aminobutyric acid (GABA) has potential in regulating hypertension. Cocoa beans are rich in GABA, but GABA is being destroyed during roasting of cocoa beans and chocolate production. This study aimed to develop GABA-enriched dark chocolate by partially replacing sugar syrup with pure GABA powder at concentrations of 0.05 (F1), 0.10 (F2), and 0.15% (F3). The chocolate samples were incorporated with GABA after the heating and melting process of cocoa butter to maintain the viability and functionality of the GABA in the final product. The effects of GABA enrichment on the quality of chocolate in terms of nutritional, physical, shelf-life, and sensorial properties were studied. The inclusion of 0.15% GABA significantly increased the GABA content and angiotensin-converting-enzyme (ACE) inhibitory effect of chocolate. The nutritional compositions of the control and GABA-enriched chocolates were almost similar. The addition of GABA significantly increased the hardness but did not affect the apparent viscosity and melting properties of chocolate. Accelerated shelf-life test results showed that all the chocolates stored at 20 and 30 °C were microbiologically safe for consumption for at least 21 days. Among the GABA-enriched chocolates, panellists preferred F2 the most followed by F3 and F1, owing to the glossiness and sweetness of F2. F3 with the highest GABA content (21.09 mg/100 g) and ACE inhibitory effect (79.54%) was identified as the best GABA-enriched dark chocolate.

## 1. Introduction

The World Health Organization defines hypertension as the chronic elevation of arterial blood pressure with the systolic blood pressure equal to or above 140 mm Hg and/or diastolic blood pressure equal to or above 90 mm Hg [1]. Hypertension is a leading cause of death globally as it is a major risk factor for stroke, cardiovascular disease, and kidney failure [2,3]. Based on the latest World Hypertension Report [4], approximately 1.13 billion people worldwide suffered from hypertension in 2019 and it is estimated to increase to 1.56 billion by 2025 [5].

The renin-angiotensin-aldosterone system is one of the main humoral regulators of the cardiovascular system that plays a significant role in regulating blood pressure. In the renin-angiotensin-aldosterone system, renin stimulates the generation of angiotensin I, which is converted to angiotensin II by zinc-dependent dicarboxypeptidase, namely angiotensin-converting enzyme (ACE), to regulate the electrolyte balance in humans. However, angiotensin II tends to narrow the blood vessels and secrete aldosterone, thus resulting in an increased blood pressure [6,7]. Several studies have reported that gamma-aminobutyric acid (GABA) could inhibit ACE through the blockade of angiotensin II formation and is promising in decreasing blood pressure [7,8].

Gamma-aminobutyric acid (GABA) is a non-proteinogenic amino acid with potent bioactive functions that occurs naturally in animals, plants, and microorganisms. GABA is synthesised in presynaptic neuron through the action of glutamic acid decarboxylase in the presence of glutamate as a precursor [9,10]. In the human central nervous system, as the principal inhibitory neurotransmitter, GABA plays a significant role in the regulation of brain metabolism and has shown physiological functions such as anti-hypertensive, anti-depressant, anti-oxidative, hypotensive, and insomnia-curing effects [7,11,12]. However, the ability of the human brain to synthesise GABA is gradually decreased with increasing age [13]. Several human studies have shown that orally administered GABA (10–20 mg/day) could decrease the blood pressure of hypertensive patients [14,15,16]. Somnipathy and depression of patients have been shown to improve through a daily intake of 26.4 mg GABA [17]. GABA can be found naturally in cocoa beans [12].

Cocoa beans are the seeds retrieved from the mature fruits of the cocoa tree known botanically as *Theobroma cacao* L. Cocoa beans are known to contain GABA naturally. Based on the study conducted by Ramos-Ruiz et al. [12], GABA content in cocoa beans originating from Africa was 35 to 93.9 mg/100 g, America 31.7 to 101.2 mg/100 g, Asia 47 to 95 mg/100 g, and Oceania 45 to 68 mg/100 g. Cocoa beans are the basic raw material for chocolate production. Chocolate is one of the most consumed and desired confectionery products across the world (2022: revenue generated from the chocolate confectionery market reached USD 1.03 trillion worldwide and is expected to increase to USD 1.12 trillion in 2027 [18]) owing to its pleasant sensory properties. Chocolate is a semi-solid suspension of fine solid particles (cocoa powder) dispersed in a continuous fat phase (cocoa butter) [19]. Chocolate can be classified into plain chocolate (dark chocolate), milk chocolate, and white chocolate, depending on its composition [20]. For instance, dark chocolate contains at least 35% of cocoa solids in total, including not less than 18% cocoa butter and 14% non-fat cocoa solids, respectively [21].

Dark chocolate, with a relatively higher cocoa content, is generally considered a good source of GABA and its consumption has been linked to a number of health benefits, including mitigation of cardiovascular disease [22], reduction of blood pressure, and improvement in cognitive health [23]. However, GABA content in dark chocolate was found to be lower than that in cocoa beans. The manufacturing of chocolate has been reported to cause the loss of GABA, such as during the fermentation of cocoa beans [24] and through the Maillard reaction during the roasting of cocoa beans [25,26]. Dark chocolate also contains other active compounds that could cause side effects. For instance, theobromine could cause headaches [27]; caffeine could cause sleep and cardiovascular problems [28]; methylxanthines could increase the risk of atrial fibrillation [29]; and histamine could contribute to migraines, heart problems, immunological sensitivity, and inflammation-like stimuli [25]. Therefore, GABA content in dark chocolate should be increased to exert beneficial health effects on consumers. Enrichment is one of the effective ways to compensate for the loss of GABA.

GABA-enriched foods have attracted considerable attention in the field of food and medicine as GABA concentration in natural animal and plant food products is too low to deliver potential health benefits to consumers. Numerous studies have examined the effects of GABA-enriched foods on hypertension in humans and experimental animals. The ingestion of GABA-enriched fermented milk (10–12 mg GABA/100 mL/day) over 12 weeks has resulted in a significant decrease in systolic blood pressure (reduced by 17.4 mm Hg) and diastolic blood pressure of 39 patients with mild hypertension (reduced by 7.2 mm Hg) [30]. Nishimura et al. [31] found that the 8-week consumption of GABA-enriched rice (150 g GABA-enriched rice/day or 16.8 mg GABA/day) has improved the blood pressure of mildly hypertensive adults. The systolic blood pressure of spontaneously hypertensive rats has been reduced from 189 mm Hg to 150 mm Hg after being fed with GABA-enriched fermented savoury rice cake (1 mg GABA/rat/day) for 10 weeks [32]. Kawakami et al. [33] also found that the ingestion of GABA-enriched brown rice (1 mg GABA/kg) for 12 weeks inhibited the elevation of blood pressure in rats. To the best of our knowledge, no studies have been carried out on GABA enrichment in dark chocolate.

Dark chocolates could be used as a promising carrier to deliver GABA because of their high acceptability by consumers. Dark chocolates are always perceived as healthier compared to other types of chocolates as they are higher in polyphenols, flavonoids, and antioxidant contents [23]. The lightweight and small size of chocolate also make them convenient for consumers to consume [20]. Therefore, this research aimed to enrich the GABA content of dark chocolate by partially replacing the sugar syrup with pure GABA powder at concentrations of 0.05, 0.10, and 0.15%. The effects of GABA enrichment on the quality of dark chocolate in terms of nutritional, textural, rheological, melting, shelf-life, and sensorial properties were also studied.

## 2. Materials and Methods

### 2.1. Ingredients

The cocoa powder, icing sugar, and lecithin were purchased at a local store in Kota Kinabalu, Malaysia. The cocoa butter was bought from Take It Global Sdn Bhd, Penang, Malaysia. GABA was acquired from NOW Foods, Bloomingdale, New York, NY, United States.

### 2.2. Preparation of Dark Chocolate

The dark chocolate was produced following the formulations specified in Table 1. Firstly, cocoa butter was melted over low heat (40–51 °C) in a double boiling setup [34]. After the cocoa butter was completely melted, sugar syrup (produced by dissolving two parts of icing sugar into one part of water) and lecithin were added, and the mixture was stirred well. Subsequently, cocoa powder and pure GABA powder were added, and the chocolate mixture was whisked until smooth and lump-free texture was obtained. The molten chocolate was removed from the pot and gently stirred until the temperature reduced to 38 °C. The molten chocolate was then transferred into a plastic mould and allowed to solidify at 16.5 °C in the refrigerator (NR-BL342PS, Panasonic, Tokyo, Japan). The dark chocolate was then wrapped with polyethylene terephthalate film with heat-sealed ends.

### 2.3. Proximate Composition

The proximate composition of the dark chocolates was determined by using the procedures established by the Association of Official Analytical Chemists [35]. The moisture content of chocolate was evaluated by oven drying 6 g of the sample at 105 °C until constant weight (AOAC Method 925.40). Soxhlet method (petroleum ether as a solvent) was used to determine the crude fat content of chocolate (AOAC Method 963.15). The ash content of chocolate was obtained by the method of incineration in a muffle furnace (KLS 30/11, Thermconcept, Bremen, Germany) at 550 °C (AOAC Method 924.05). The crude protein content was measured by estimating the nitrogen content in the chocolate using the Kjeldahl method (AOAC Method 970.22, nitrogen-to-protein conversion factor: 6.25). The crude fibre content of the chocolate was determined using an automated fibre analyser (Fibretherm FT12, C. Gerhardt GmbH & Co., Königswinter, Germany) (AOAC Method 962.09). The carbohydrate content was calculated by subtracting the total values of moisture, crude protein, fat, ash, and fibre of the sample from 100 (Equation (1)). The energy content provided by the chocolate was calculated by adding up the calories provided by the protein, carbohydrate, and fat (Equation (2)).

Carbohydrate (%) = 100 − (Moisture + Crude protein + Total fat + Ash + Total dietary fibre)(1)

(2) $$\mathrm{Energy}\;\left(\mathrm{kcal}\right)=\left(\%\;\mathrm{protein}\;\times 4\right)+\left(\%\;\mathrm{carbohydrate}\;\times 4\right)+\left(\%\;\mathrm{fat}\;\times 9\right)$$

where 4 is the energy conversion factor for protein and carbohydrate, and 9 is the energy conversion factor for fat.

### 2.4. Quantification of GABA in Dark Chocolate Using HPLC

The GABA content of the dark chocolates was measured according to the method proposed by Dala-Paula et al. [25]. To extract GABA from the dark chocolate, the prepared chocolate (5 g) was grounded and homogenised with trichloroacetic acid (TCA, 20 mL, 6% *w*/*v*, R&M Chemicals, Selangor, Malaysia) for 3 min. After being subjected to centrifugation (7000× *g*, 10 min, 4 °C), the supernatant was filtered (Whatman, No. 2, Maidstone, UK) and the final volume of the filtrate was brought up to 25 mL with TCA. Both the standard and extract (diluted filtrate) solutions were then subjected to the benzoylation process. The standard (50 µL) and extract solution (2 mL) were mixed with sodium hydroxide (NaOH, 2 mL, 2 M, R&M Chemicals, Selangor, Malaysia) followed by benzoyl chloride (10 µL, Sigma-Aldrich, St. Louis, MO, USA). After being kept at room temperature (25 °C) for 20 min, the benzoylation processes of GABA were stopped by the addition of saturated sodium chloride (NaCl, 2 mL, Merck, Frankfurt, Germany). Subsequently, diethyl ether (4 mL, R&M Chemicals, Selangor, Malaysia) was added and the solutions were subjected to centrifugation (7000× *g*, 10 min, 4 °C). The upper layer, post-centrifugation, was transferred to a universal bottle, dried under a laminar flow (Model AHC-4A1-ESCO, Changi, Singapore), and dissolved in methanol (500 µL, HPLC grade, JT Baker, Phillipsburg, NJ, United States) for semi-preparative high-performance liquid chromatography with diode array detection (Shimadzu LC-6AD instrument, Kyoto, Japan). Chromatographic separation was carried out on a C18 analytical column (15.9 × 4.6 mm, Thermo Scientific Hypersil Gold, Shimazdu, Kyoto, Japan) maintained at 35 °C and a gradient elution consisting of water (A) and methanol (B) (at 0.00–2.00, 4.00, 5.50, 10.00, 12.00, 15.50, 15.51–19.00, and 19.01 min, with solvent A at 89, 83, 69, 67.5, 53.5, 45, 0, and 89%, respectively) with a flow rate of 0.4 mL/min. The sample injection volume was 1 μL and the detection wavelength was set at 230 nm. The concentration of GABA in the dark chocolate was then quantified by fitting the peak areas obtained into the linear regression equation of the GABA standard curve prepared from known concentrations of GABA.

### 2.5. Angiotensin-Converting-Enzyme (ACE) Inhibitory Activity

The ACE inhibitory activity of the dark chocolates was determined using the methodology proposed by Chen et al. [36]. HHL and ACE solution were prepared by dissolving the hippuryl-L-histidyl-L-leucine (HHL, Sigma-Aldrich, St. Louis, MO, USA) and rabbit lung acetone powder (HHL, Sigma-Aldrich, St. Louis, MO, USA) in borate suffered saline (100 mM sodium borate buffer (0.5 M, pH 8.5, Thermo Scientific, Calsbad, CA, USA), 300 mM NaCl (Merck, Frankfurt, Germany), pH 8.3), respectively. Melted dark chocolate (50 µL) was pre-incubated in HHL solution (50 µL, 10 mM) at 37 °C for 2 min. Subsequently, ACE (50 µL, 0.010 U/mL) solution was added, and the mixture was further incubated at 37 °C for 30 min. After the reaction was terminated by heating the mixture in a water bath at 85 °C for 10 min, the mixture (20 µL) was diluted with deionized water (150 µL). The resulting mixture was then analysed by using a high-performance liquid chromatography (Shimadzu LC-6AD instrument, Kyoto, Japan) equipped with a C18 analytical column (15.9 × 4.6 mm, Thermo Scientific Hypersil Gold, Shimazdu, Kyoto, Japan). The temperature of the column was maintained at 30 °C. Elution was isocraticed with acetonitrile (≥99.9%, Sigma-Aldrich, St. Louis, MO, USA)/water (75:25, *v*/*v*) containing 0.1% TCA at a constant flow rate of 1.5 mL/min. The eluent was monitored at 228 nm and the ACE inhibitory activity was calculated using Equation (3) below:

ACE inhibitory activity (%) = (C − S)/(C − A) × 100(3)

where C, S, and A are the chromatography peak area of borate suffered saline (control), tested sample, and ACE solution, respectively.

### 2.6. Textural Properties: Hardness

The hardness values of the dark chocolates were determined by using the penetration test method proposed by McGill and Hartel [37], with slight modification. A texture analyser (TA. XT Plus, Stable Micro Systems Ltd., Surrey, UK) equipped with a 500 N load cell and needle geometry was used to measure the hardness values of the chocolates. The hardness was reported as the maximum penetrating force (N) required for the needle to penetrate through the chocolate (35 mm × 6 mm, depth 15 mm) at 20 °C, over a distance of 5 mm at a pre-test speed of 2 mm/s, test speed of 2 mm/s, and post-test speed of 10 mm/s.

### 2.7. Rheological Measurements: Apparent Viscosity

The rheological properties of the dark chocolates were determined using the method proposed by Cahyani et al. [38]. Before the analysis, chocolates (15 g) were placed in a plastic test tube and melted in a water bath (Memmert WB 14, Schwabach, Germany) at 40 °C. The viscosity of the molten chocolate was then measured by using a viscometer (DV-E, Brookfield, Middleborough, MA, USA) with spindle number 7 at a rotating speed of 10, 12, 20, 30, 50, 60, and 100 rpm. The shear stress (τ) and shear rate (γ) of the molten chocolate were calculated from the viscometric data (dial reading) obtained using the Mitschka methodology (Equations (4)–(5)). The apparent viscosity (η) of the molten chocolate was then obtained by dividing shear stress with shear rate (Equation (6)):

τ = dial reading × 8.4(4)

(5) $$\gamma =\mathrm{rpm}\;\times {\mathrm{Kn}}_{\gamma }$$

(6) η=τ/γ

where 8.4 is the conversion factor for spindle number 7, Kn_γ_ is the shear rate consistency obtained by matching the spindle number with the flow behaviour index value (n), and n is the linear slope in the plot of the ln τ versus ln rpm.

### 2.8. Melting Properties

The melting properties of the dark chocolates were studied using differential scanning calorimetry (DSC 1, Mettler Toledo, Schwerzenbach, Switzerland) equipped with a data station. The dark chocolate sample (5 mg) was loaded into a hermetically sealed aluminium pan and heated from 15 to 55 °C (expected range for melting profile of chocolate) in a nitrogen stream at 5 °C/min, using an empty pan as reference [39]. The onset temperature (T_onset_), end temperature (T_end_), peak temperature (T_peak_), and melting enthalpy (ΔH_melt_) were computed by the software.

### 2.9. Accelerated Shelf-Life Test: Microbial Analysis

The accelerated shelf-life test of dark chocolates was designed based on [40]. Freshly produced chocolates were heat-sealed in polyethylene bags and kept in two different incubators (Binder BD-53, Binder GmbH, Tuttlingen, Germany) maintained at 20 and 30 °C with a relative humidity level of 80% for 3 weeks. The microbial analysis was performed on the chocolates on days 1, 6, 8, 13, 15, and 21 of storage. Plate count agar (PCA, Merck, Frankfurt, Germany) and potato dextrose agar (PDA, Merck, Frankfurt, Germany) were used to enumerate the total viable count and yeast and mould count, respectively. The dark chocolate sample (10 g) was crushed and homogenised with sterilized peptone water (90 mL, Merck, Frankfurt, Germany) using a stomacher (Interscience Bag Mixer, Saint Nom, France). Subsequently, the homogenised solution was subjected to a 10-fold serial dilution with sterilized peptone water (Merck, Frankfurt, Germany). An aliquot of appropriate dilution was then inoculated onto the PCA (Merck, Frankfurt, Germany) and PDA (Merck, Frankfurt, Germany) plates using the pour plate technique. After incubation (37 °C, 48 h), the grown colonies were then counted, and the counts were reported as colony forming units per gram of the chocolate sample (CFU/g).

### 2.10. Hedonic Test

A hedonic test was performed to select the dark chocolate formulation with the most acceptable sensory characteristics. A total of 55 well-trained panellists (18–60 years old, students and employees of the Faculty of Food Science and Nutrition, University Malaysia Sabah, Sabah, Malaysia) were recruited to evaluate the glossiness, hardness, melting rate, sweetness, and overall acceptability of the chocolate samples by using a 7-point hedonic scale (1 = dislike extremely, 2 = dislike moderately, 3 = dislike slightly, 4 = neither like nor dislike, 5 = like slightly, 6 = like moderately, and 7 = like extremely). The chocolate samples (5 g) were served in transparent plastic containers coded with 3-digit random numbers. Mineral water was provided for panellists who were instructed to rinse their mouths before and between each evaluation.

### 2.11. Statistical Analysis

All the analyses were performed in triplicate and the results from quantification of GABA, ACE inhibitory activity, hardness, apparent viscosity, and melting properties obtained in this study were statistically analysed by one-way analysis of variance (ANOVA) with Tukey’s honestly significant difference (HSD) test for post-hoc comparisons using the software IBM SPSS Statistics version 27 (IBM, Chicago, IL, USA). Kruskal–Wallis test was used to analyse the results from the sensory test in the present study [41]. A probability level of < 0.05 was considered statistically significant in all comparisons.

## 3. Results and Discussion

### 3.1. Proximate Composition

The nutritional content of the control and GABA-enriched dark chocolates is shown in Table 2.

The moisture contents of all the dark chocolates are presented in Table 2. Dark chocolates enriched with 0.10% (F2) and 0.15% GABA (F3) had significant (*p* < 0.05) lower moisture contents than the control dark chocolate (without GABA enrichment, C). Among the GABA-enriched dark chocolates, the moisture content of F1 with 0.05% GABA was the highest, followed by F2 and F3, which had the lowest moisture content. The decrement in the moisture contents could be attributed to the decrease of sugar syrup used in the formulation. Sucrose syrup was used as a sweetener in the preparation of dark chocolates. The monosaccharides presented in the sucrose syrup caused difficulties in the drying of chocolate and hence resulted in chocolate with higher moisture content [37]. A similar finding was reported by [42], who also observed that the moisture content of chocolate increased with the addition of sucrose syrup.

As presented in Table 2, F3 had the highest protein content (6.43%), followed by F2 (6.41%), F1 (6.39%), and C (6.37%). Although no significant differences (*p* > 0.05) were observed, the protein contents of the dark chocolates increased with the concentration of GABA incorporated. The increment in the protein content could be attributed to the non-protein nitrogen contributed by GABA. As the Kjeldahl method could not differentiate protein nitrogen from non-protein nitrogen [43], the incorporation of GABA eventually increased the total nitrogen content and consequently resulted in higher protein content.

The dark chocolate with the highest fat content was F1 (31.10%), followed by F3 (30.80%), C (29.48%), and F2 (29.47%), which had the lowest fat content. No significant differences were observed between the fat contents of the control (C) and GABA-enriched chocolates (F1, F2, and F3). This result was expected as the amounts of cocoa butter used were similar in all the formulations. Cocoa butter is the key contributor to the fat composition of chocolate. The fat composition of conventional dark chocolates is normally between 30 and 40% [44].

The fibre content in all the dark chocolates (C, F1, F2, and F3) ranged between 14.70 and 14.77%, with no significant differences observed. The results implied that the replacement of sugar syrup with GABA did not affect the fibre content of chocolates. The fibre content in the chocolates was attributed to the presence of cocoa powder in the formulation. Fibre is a major constituent of cocoa powder [45]. According to the permitted nutrition claim listed in the Annex of Regulation (EC) No 1924/2006 [46], a food product with at least 6 g of fibre per 100 g can be claimed as “high in fibre”. Therefore, all the dark chocolates developed in this work can be characterised as high-fibre food products.

Ash content could reflect the total mineral content in the chocolate [47]. The ash contents of all the dark chocolates were not significantly different from one another (Table 2). The incorporation of GABA did not affect the ash contents of the chocolates. The ash contents of the chocolates were mainly contributed by the cocoa powder. Cocoa powder is rich in minerals such as sodium (Na), potassium (K), magnesium (Mg), calcium (Ca), phosphorus (P), iron (Fe), and zinc (Zn). A previous study reported that 100 g of cocoa powder consisted of 5.27–11.5 mg Na, 2.27–3.97 g K, 3.11–5.02 g Mg, 0.10–0.22 g Ca, 0.41–0.51 g P, 0.01–0.13 g Fe, and 3.77–7.03 mg Zn [48].

The carbohydrate content of the dark chocolates ranged from 29.73 (F1) to 31.49% (F2). No significant difference (*p* > 0.05) was found between the carbohydrate content of the dark chocolates. The results indicated that the partial replacement of sugar syrup with GABA did not change the carbohydrate content of the dark chocolates. The cocoa powder and sugar syrup were the major carbohydrates in the formulation [45]. The reduction in the concentration of added sugar syrup (from 45 to 44.85%) was presumed to be too low to exert significant effects.

As shown in Table 2, the calorie content of the dark chocolates developed in this study ranged from 415.97 to 424.47 kcal, and there was no significant difference (*p* > 0.05) among the chocolates with and without the enrichment of GABA. Dark chocolate has been classified as a high-calorie food owing to its high fat and sugar content. For instance, the USDA Food Composition Database [49] stated that the calorie contents of dark chocolates (45–85% cocoa solids) ranged from 546 to 598 kcal/100 g. The calorie contents of the dark chocolates developed in this study (54.50% cocoa solids) were relatively lower. This could be attributed to the lower carbohydrate content of the chocolates developed.

### 3.2. GABA Content

Chocolate was proven to have GABA naturally from the cocoa bean [12]. However, the chocolate-making process has been reported to reduce the GABA content in chocolate [24,25]. According to the Code of Federal Regulations (40 CFR Part 180.1188), GABA is permitted to be used as a food ingredient, with no limitations cited for its use, by adhering to good agricultural practices [50]. Similarly, the safe use of GABA in food has also been reported by the Joint FAO/WHO Expert Committee on Food Additives (JECFA, Rome, Italy) [51]. A dose of 50–3000 mg GABA/day (<750 mg per dose) has been suggested by the Natural and Non-prescription Health Products Directorate (NNHPD) for cognitive performance, but health consultation is necessary when a dose of 300 mg GABA/day is kept for more than 4 weeks [52]. In Malaysia, the June 2022 edition of the Monthly Index of Medical Specialities (MIMS) suggested that the dosages of GABA for the treatment of mental deficiency were 250–500 and 125–250 mg (three times daily) for adults and children, respectively [53]. Therefore, GABA levels of 50 mg/100 g (0.05%), 100 mg/100 g (0.10%), and 150 mg/100 g (0.15%), which were used to develop GABA-enriched dark chocolate in this study, are within the safety limit to guarantee the consumer safety; hence, the conduct of toxicity study is not necessary in this case. The quantification of GABA in the control and GABA-enriched dark chocolates was conducted by using HPLC. Table 3 presents the GABA contents of all the chocolates in this study.

The concentration of GABA in the dark chocolates was observed to increase gradually with the amount of GABA incorporated: C (GABA incorporated = 0%; concentration of GABA in chocolate = 8.23 mg/100 g) < F1 (0.05%; 11.60 mg/100 g) < F2 (0.10%; 16.96 mg/100 g) < F3 (0.15%; 21.09 mg/100 g). Although all the GABA-enriched chocolates had higher GABA content than C, a significant increment (*p* < 0.05) was only observed in F3 with 0.15% GABA. The GABA contents in all the GABA-enriched chocolates were found to be lesser than the amount of GABA added initially during the production. For instance, after incorporating 5 mg/100 g (0.05%) of GABA, the GABA content in F1 was observed to increase 3.37 mg/100 g (as compared with C). By comparing the GABA content of the control chocolate (8.23 mg/100 g) with the GABA contents of cocoa bean (35–95 mg/100 g) [12] and cocoa powder (82.14 mg/100 g) [24] from the literature, GABA tended to be lost during the processing of chocolate. The reduction of GABA content in the GABA-enriched dark chocolates could be attributed by the Maillard reaction between GABA and sugar [25,26]. GABA was observed to have a great chemical reactivity with carbonyl compounds (sugar degradation products) during the Maillard reaction [54]. The results suggest that enrichment is one of the most efficient ways to compensate for the reduction of GABA.

### 3.3. Angiotensin-Converting-Enzyme (ACE) Inhibitory Activity

GABA and GABA-enriched food have been reported to show ACE inhibitory activities [7,8]. Table 4 shows the inhibitory effects of the control and GABA-enriched dark chocolates on ACE.

As shown in Table 4, the ACE inhibition of the dark chocolates was observed to increase significantly (*p* < 0.05) in a concentration-dependent manner as the GABA concentration increased. The results showed that the GABA enrichment could enhance the ACE-inhibitory activities of dark chocolates effectively. GABA inhibits the ACE activities by binding itself to the binding sites of the ACE through hydrophobic interaction and zinc ion chelation [7]. When the concentration of GABA increased, more binding sites of ACE were occupied and hence resulted in higher inhibition effects. Similar findings were reported by Tu et al. [7], Guiyun et al. [8], and Ji et al. [55], who worked on GABA-enriched mulberry leaves, buckwheat powder, and coffee leaves, respectively. A significant correlation (r = 0.965, *p* < 0.01) was found between the GABA content and ACE inhibition of GABA-enriched mulberry leaves [7]. The ACE inhibition of buckwheat powder (increment of GABA content: 2.22 mg/g; increment of ACE inhibition: 87.80%) and coffee leaves (increment of GABA content: 79%; increment of ACE inhibition: 79.00%) was positively impacted by the increased GABA content [8,55].

### 3.4. Hardness

Hardness is one of the important features of chocolate that could affect the sensory perception of consumers [39]. Table 5 shows the hardness of the dark chocolates in this study.

The replacement of sugar syrup with GABA significantly (*p* < 0.05) increased the hardness of chocolates, with the control chocolate (0% GABA) that had the lowest hardness (468.10 g) < F1 (0.05% GABA, 618.57 g) < F2 (0.10% GABA, 845.36 g) < F3 that contained the highest amount of GABA (0.15% GABA) and showed the highest hardness (923.41 g). The increment in the hardness of chocolates could be attributed to the interactions between the crystallised continuous fat phase (cocoa butter) and the dispersed solid particles (pure GABA powder). The incorporated pure GABA powder could interact with the free fat in chocolate, re-crystallise the fat crystal into a polymorphic structure with higher stability and compactness, and thus result in a harder texture [56]. A similar result was reported by Lončarević et al. [57], who reported an increase in the hardness of chocolate when the concentration of solid particles in the formulation increased.

### 3.5. Apparent Viscosity

The rheological properties of chocolate are closely related to the chocolate quality. For instance, the order and contact rate of chocolate between the tongue and the mouth influence the taste of chocolate perceived by consumers [20]. The apparent viscosities of all the dark chocolates are shown in Table 6.

As can be seen in Table 6, all the dark chocolates exhibited lower apparent viscosity at a higher rotating speed or shear rate, indicating that all the chocolates showed pseudoplastic and shear-thinning behaviour. The shear-thinning behaviour described the rheological properties of chocolate, in which the structure of chocolate could remain stable at rest but be progressively broken down when shear force was applied [58]. Our results were aligned with [58,59], who also reported on the shear-thinning behaviour of chocolates. The incorporation of GABA did not negatively affect the shear-thinning nature of chocolate.

At a rotating speed of 10 rpm, C displayed the highest (*p* > 0.05) apparent viscosity (34.17 mPa∙s), followed by F1 (30.24 mPa∙s), F2 (19.44 mPa∙s), and F3 (17.03 mPa∙s). The apparent viscosities of chocolates decreased with increasing levels of sugar replacement. The results indicated that sugar syrup plays a prominent role in dictating the viscosity of chocolate. Owing to the hydrophilic and hygroscopic nature of sugar, it was very difficult to dry and therefore caused an increment in the moisture of chocolates. In the presence of water, when the chocolate was melted, the interactions between particles increased, subsequently increased the frictional force between molten chocolate and the spindle limit, and thus resulted in higher dial reading or higher viscosity results [60].

### 3.6. Melting Properties

The melting profiles of the control dark chocolate and dark chocolates enriched with varying amounts of GABA are shown in Table 7.

Table 7 shows that there was no significant difference between the chocolates in terms of melting profiles (T_onset_, T_peak_, T_end_, and ΔH_melt_). Peak onset (T_onset_), peak maximum (T_peak_), offset temperature (T_end_), and enthalpy of melting (ΔH_melt_) corresponds to the temperatures at which the chocolate starts to melt, melts at the fastest rate, melts completely, and the energy required to melt chocolate, respectively [39]. Based on the DSC thermograms (results not shown), all the chocolates displayed a single endothermic transition between the temperature of 15 and 55 °C. These indicated that crystallization phenomena had taken place in the chocolates. From the DSC thermograms, the heat flow consistently rose to T_onset_ and increased extremely until T_peak_ was obtained. Subsequently, the heat flow decreased until it remained consistent (T_end_). The results implied that all the chocolates were completely melted, and the incorporation of GABA did not affect the melting behaviour of chocolate.

### 3.7. Accelerated Shelf-Life Test: Microbial Analysis

In this study, an accelerated shelf-life test was conducted to predict the shelf-life of the dark chocolates in a shorter period. Table 8 shows the microbial count from the microbiological analysis conducted on day 1, 6, 8, 13, 15, and 21 at 20 and 30 °C. Based on the results obtained, the total bacteria, yeast, and mould counts in all the chocolates rose on the 13th day of storage. On the 21st day, F3 showed the highest total bacteria, yeast, and mould counts at both storage temperatures of 20 and 30 °C among all the chocolates. This could be attributed to the higher moisture content of F3 (17.13%) compared to other chocolates (15.82–16.59%) (Table 2). F3 with higher water availability provided a more favourable condition for the growth of bacteria, yeast, and mould [61]. The high moisture content of chocolates, which was contributed by the sugar syrup, is identified as the limiting criteria in the shelf-life test of the present study. The modification of dark chocolate formulation with lower moisture content has also been suggested as a feasible approach to obtain a reliable and realistic representation of actual shelf life of the GABA-enriched dark chocolate. Since no bacteria, yeast, and mould was detected on the 1st day of storage, the possible contamination during the production of chocolates can be eliminated [62]. According to the microbiological quality guideline limit set by the US Food and Drug Administration [63] and the International Commission on Microbiological Specifications for Foods (ICMSF) [64], at the time of consumption, the total bacteria and yeast and mould count of chocolates must not exceed 10^3^ CFU/g and 10 CFU/g, respectively. Therefore, all the chocolates in the present study were microbiologically safe for consumption for at least 21 days of storage at both 20 and 30 °C.

### 3.8. Hedonic Test

In this study, a hedonic test was conducted to study the consumers’ preferences on the GABA-enriched chocolates. The sensory scores for the glossiness, hardness, melting rate, sweetness, and overall acceptability of the chocolates are shown in Table 9.

Consumers’ preferences towards chocolate appear to depend on their first sight at its appearance, where high-quality dark chocolate should possess a dark brown colour with a glossy surface appearance [65]. Among the chocolates, F2 (4.93) obtained the highest mean appearance score, followed by C (4.91), F1 (4.80), and F3 (4.73), which scored the lowest. Since there were no significant differences (*p* > 0.05) between the appearance scores obtained for the chocolates, we can conclude that the addition of GABA did not show notable effects on the glossiness of the chocolate. The glossier appearance of the chocolates was closely related to their fat composition. Cocoa butter, the major fat ingredient for chocolate making, contains a large number of fatty acids such as arachidic acid, linoleic acid, palmitic acid, oleic acid, palmitoleic acid, and stearic acid. A previous study has shown that chocolate bars with 15% (*w*/*w*) cocoa butter displayed a glossy surface appearance [65]. Therefore, the chocolates (27% (*w*/*w*) cocoa butter) in this work were expected to show a glossy appearance.

Hardness is one of the dominant attributes of chocolate, which influences mainly the mastication process and the sensation associated with melting [66]. For the attribute of hardness, F3 obtained the highest mean score (4.81), followed by F2 (4.51), C (4.46), and F1 (4.30), with no significant differences (*p* > 0.05) observed between the chocolates. The results indicated that F1 was the least likable and acceptable by panellists in terms of hardness. According to the panellists, F1 was soft to be chewed and failed to give a sensation of crispiness as F3 did during the first bite. Meanwhile, even though the hardness of F2 was acceptable, the crunchy sensation perceived was not desired. This could be due to the lecithin ingredient, which might have not been completely dissolved in the molten chocolate during the making of chocolate.

Chocolate is known to have a unique mouthfeel owing to the narrow melting range of cocoa butter. In terms of mouthfeel, F1 obtained the highest mean score (5.25), followed by C (5.07), F2 (4.82), and F3 (4.55), with no significant differences (*p* > 0.05) observed among all the chocolates. Although the results were not significant, most of the panellists preferred the mouthfeel displayed by F1. As can be seen in Table 8, F1 was the most likeable and acceptable in terms of its mouthfeel. This could be due to the melting properties of F1. The low melting point (T_peak_) and ΔH_melt_ allow F1 to melt and dissolve in the mouth easier compared to other chocolates. By interpreting the hardness and mouthfeel attributes tested, the panellists were found to prefer dark chocolate with good melting properties but hard in texture before masticating it.

Taste is one of the important determinants of chocolate acceptance. The mean taste scores of the chocolates ranged from 4.70 to 5.03, with F2 scoring the highest. Although there were no significant differences (*p* > 0.05) found in the scores obtained, panellists indicated their preferences for the bittersweet taste of F2, as compared to other chocolates. According to the formulations designed, C consists of the highest amount of syrup. Meanwhile, the sugar content in F1, F2, and F3 was gradually reduced by 0.05% and substituted by GABA pure powder. However, based on the feedback received, panellists claimed that the bittersweetness of F1 was too bland. These were due to the differences in the taste perception of panellists [67].

No significant difference (*p* > 0.05) was found between the chocolates in terms of the overall acceptability. According to the mean scores, most of the panellists preferred the control chocolate (5.60) compared to the GABA-enriched chocolates (F2 (5.51), F3 (5.40), and F1 (5.37)). Among the GABA-enriched chocolates, F2 was the most preferred chocolate by the panellists. This could be attributed to the glossiness and sweetness of F2.

There was no significant difference between the sensory scores of the GABA-enriched dark chocolates for all sensory attributes tested (*p* > 0.05). This indicated that the addition of GABA did not show notable effects on the sensory acceptance of the chocolate.

## 4. Conclusions

The present work demonstrated the enrichment of dark chocolate with pure GABA powder at concentrations of 0.05, 0.10, and 0.15%. The incorporation of GABA did not show any significant effects on the rheological, melting, shelf-life, and sensorial properties but caused an increment in the moisture, protein content, and hardness of dark chocolate. The GABA content and ACE inhibitory activity of the dark chocolate were significantly enhanced with the incorporation of 0.15% GABA. Overall, F3 is the best GABA-enriched dark chocolate in this study, owing to its highest GABA content (21.09 mg/100 g) and ACE inhibitory effect (79.54%). The chocolate production process significantly reduces the content of GABA and the only way to tackle this issue is by enriching the product with 0.15% of GABA after the double boiling process of cocoa butter to achieve 2.5 times higher amount of GABA as compared to the control. Therefore, 0.15% of GABA enrichment in chocolate product after the cocoa butter melting step was deemed to be an effective method to compensate the loss of GABA throughout the chocolate production. GABA enrichment could improve the blood pressure regulating effect of dark chocolate. From the economic point of view, the formulated and value-added GABA-enriched dark chocolate could diversify the functional food market.

## Figures and Tables

**Table 1:** Formulation of the control and GABA-enriched dark chocolates.

Formulations	Ingredients (% *w*/*w*)				
	Sugar Syrup	Cocoa Powder	Cocoa Butter	GABA	Lecithin
C	45.00	27.50	27.00	0.00	0.50
F1	44.95	27.50	27.00	0.05	0.50
F2	44.90	27.50	27.00	0.10	0.50
F3	44.85	27.50	27.00	0.15	0.50

C = control sample (without GABA); F1 = dark chocolate enriched with 0.05% GABA; F2 = 0.10% GABA; F3 = 0.15% GABA.

**Table 2:** Nutritional content of control and GABA-enriched dark chocolates.

Formulations	Nutritional Content (%)						
	Moisture	Protein	Fat	Fibre	Ash	Carbohydrate	Calorie (kcal)
C	15.89 ± 0.06 ^a^	6.37 ± 0.01 ^c^	29.48 ± 0.77 ^a^	14.73 ± 0.23 ^a^	2.24 ± 0.06 ^a^	31.29 ± 0.87 ^a^	415.97 ± 3.48 ^a^
F1	15.78 ± 0.04 ^ab^	6.39 ± 0.01 ^bc^	31.10 ± 0.98 ^a^	14.70 ± 0.16 ^a^	2.31 ± 0.14 ^a^	29.73 ± 0.76 ^a^	424.34 ± 5.88 ^a^
F2	15.61 ± 0.07 ^b^	6.41 ± 0.01 ^ab^	29.47 ± 0.74 ^a^	14.77 ± 0.9 ^a^	2.25 ± 0.13 ^a^	31.49 ± 0.86 ^a^	416.77 ± 3.34 ^a^
F3	15.34 ± 0.10 ^c^	6.43 ± 0.01 ^a^	30.80 ± 1.05 ^a^	14.73 ± 0.13 ^a^	2.32 ± 0.09 ^a^	30.38 ± 1.26 ^a^	424.47 ± 5.32 ^a^

Mean ± SD values (*n* = 3) with a different superscript ^a–c^ in the same column were significantly different (Tukey’s HSD, *p* < 0.05). C = control sample (without GABA); F1 = dark chocolate enriched with 0.05% GABA; F2 = 0.10% GABA; F3 = 0.15% GABA.

**Table 3:** GABA content of control and GABA-enriched dark chocolates.

Formulations	Concentration of GABA (mg/100 g)
C	8.23 ± 0.14 ^b^
F1	11.60 ± 0.51 ^b^
F2	16.96 ± 2.11 ^ab^
F3	21.09 ± 4.99 ^a^

Mean ± SD values (*n* = 3) with a different superscript ^a,b^ in the same column were significantly different (Tukey’s HSD, *p* < 0.05). C = control sample (without GABA); F1 = dark chocolate enriched with 0.05% GABA; F2 = 0.10% GABA; F3 = 0.15% GABA.

**Table 4:** Inhibitory effects of control and GABA-enriched dark chocolates on angiotensin-converting enzyme (ACE).

Formulations	ACE Inhibition (%)
C	54.41 ± 0.90 ^d^
F1	62.20 ± 0.89 ^c^
F2	71.02 ± 1.14 ^b^
F3	79.54 ± 1.53 ^a^

Mean ± SD values (*n* = 3) with a different superscript ^a–d^ in the same column were significantly different (Tukey’s HSD, *p* < 0.05). C = control sample (without GABA); F1 = dark chocolate enriched with 0.05% GABA; F2 = 0.10% GABA; F3 = 0.15% GABA.

**Table 5:** Hardness of control and GABA-enriched dark chocolates.

Formulations	Hardness (g)
C	468.10 ± 10.77 ^d^
F1	618.57 ± 7.93 ^c^
F2	845.36 ± 20.07 ^b^
F3	923.41 ± 41.81 ^a^

Mean ± SD values (*n* = 3) with a different superscript ^a–d^ in the same column were significantly different (Tukey’s HSD, *p* < 0.05). C = control sample (without GABA); F1 = dark chocolate enriched with 0.05% GABA; F2 = 0.10% GABA; F3 = 0.15% GABA.

**Table 6:** Apparent viscosity of control and GABA-enriched dark chocolates.

Formulations	Apparent Viscosity (10^4^ mPa∙s)						
	10 rpm	12 rpm	20 rpm	30 rpm	50 rpm	60 rpm	100 rpm
C	34.17 ± 2.61 ^a^	14.63 ± 0.79 ^a^	6.95 ± 0.45 ^a^	2.89 ± 0.18 ^a^	1.57 ± 0.43 ^a^	0.56 ± 0.06 ^a^	0.31 ± 0.06 ^a^
F1	30.24 ± 0.48 ^a^	14.76 ± 1.27 ^a^	7.07 ± 0.097 ^a^	2.80 ± 0.20 ^a^	1.44 ± 0.07 ^a^	0.47 ± 0.01 ^a^	0.19 ± 0.01 ^a^
F2	19.44 ± 4.50 ^a^	14.42 ± 3.14 ^a^	4.99 ± 11.01 ^a^	2.20 ± 0.73 ^a^	0.92 ± 0.26 ^a^	0.55 ± 0.17 ^a^	0.24 ± 0.08 ^a^
F3	17.03 ± 0.39 ^a^	12.92 ± 0.71 ^a^	4.97 ± 0.44 ^a^	1.75 ± 0.19 ^a^	0.74 ± 0.08 ^a^	0.61 ± 0.02 ^a^	0.29 ± 0.01 ^a^

Mean ± SD values (*n* = 3) with a similar superscript ^a^ in the same column were not significantly different (Tukey’s HSD, *p* > 0.05). C = control sample (without GABA); F1 = dark chocolate enriched with 0.05% GABA; F2 = 0.10% GABA; F3 = 0.15% GABA.

**Table 7:** Melting properties of control and GABA-enriched dark chocolates.

Formulations	T_onset_ (°C)	T_peak_ (°C)	T_end_ (°C)	ΔH_melt_ (J/g)
C	30.65 ± 0.69 ^a^	33.45 ± 0.50 ^a^	35.70 ± 0.48 ^a^	22.35 ± 1.32 ^a^
F1	31.00 ± 0.15 ^a^	33.47 ± 0.21 ^a^	35.42 ± 0.61 ^a^	20.48 ± 8.41 ^a^
F2	31.03 ± 0.15 ^a^	33.53 ± 1.09 ^a^	35.82 ± 0.75 ^a^	30.29 ± 1.55 ^a^
F3	31.97 ± 1.78 ^a^	33.61 ± 0.16 ^a^	35.04 ± 0.45 ^a^	24.33 ± 0.19 ^a^

Mean ± SD values (*n* = 3) with a similar superscript ^a^ in the same column were not significantly different (Tukey’s HSD, *p* > 0.05). C = control sample (without GABA); F1 = dark chocolate enriched with 0.05% GABA; F2 = 0.10% GABA; F3 = 0.15% GABA.

**Table 8:** Bacteria, yeast, and mould count of control and GABA-enriched dark chocolates.

**Formulations**	**Storage Period (Day)**											
	**1**		**6**		**8**		**13**		**15**		**21**	
	20 °C	30 °C	20 °C	30 °C	20 °C	30 °C	20 °C	30 °C	20 °C	30 °C	20 °C	30 °C
	**Bacteria Count (CFU/g)**											
C	-	-	-	-	TNTC	-	-	7.5×10^−3^	2.1 × 10^−1^	1.2 × 10^−3^	4.4 × 10^−1^	1.3 × 10^−1^
F1	-	-	TNTC	-	-	TNTC	2.9 × 10^−2^	4.5 × 10^−3^	7.7 × 10^−2^	1.2 × 10^−2^	8.7 × 10^−2^	2.3 × 10^0^
F2	-	-	-	-	-	-	2.9 × 10^−2^	TNTC	1.1 × 10^0^	1.2 × 10^−2^	2.0 × 10^0^	2.1 × 10^−1^
F3	-	-	-	-	1.4 × 10^−2^	5.8 × 10^−2^	1.7 × 10^−1^	1.6 × 10^−2^	2.3 × 10^−1^	1.2 × 10^0^	2.3 × 10^0^	2.7 × 10^0^
	**Yeast and mould count (CFU/g)**											
C	-	-	TNTC	TNTC	TNTC	-	-	-	1.7 × 10^0^	6.7 × 10^−3^	2.3 × 10^0^	9.5 × 10^−1^
F1	-	-	TNTC	-	TNTC	TNTC	1.7 × 10^−2^	2.4 × 10^−3^	1.7 × 10^−2^	1.1 × 10^−2^	1.0 × 10^−1^	2.0 × 10^0^
F2	-	-	TNTC	TNTC	TNTC	-	9.5 × 10^−2^	-	1.1 × 10^−1^	1.4 × 10^−2^	2.1 × 10^0^	2.0 × 10^0^
F3	-	-	TNTC	-	9.2 × 10^−3^	-	1.4 × 10^−1^	6.6 × 10^−3^	1.4 × 10^−1^	8.4 × 10^−2^	2.3 × 10^0^	2.9 × 10^0^

C = control sample (without GABA); F1 = dark chocolate enriched with 0.05% GABA; F2 = 0.10% GABA; F3 = 0.15% GABA; TNTC = too numerous to count; - = no visible colony.

**Table 9:** Hedonic sensory scores of control and GABA-enriched dark chocolates.

Formulations	Sensory Attributes				
	Appearance: Glossiness	Texture: Hardness	Mouthfeel: Melting Rate	Taste: Bittersweetness	Overall Acceptability
C	4.91 ± 1.25 ^a^	4.46 ± 1.94 ^a^	5.07 ± 0.24 ^a^	5.02 ± 0.19 ^a^	5.60 ± 0.19 ^a^
F1	4.80 ± 1.41 ^a^	4.30 ± 0.25 ^a^	5.25 ± 0.21 ^a^	4.96 ± 0.21 ^a^	5.37 ± 0.15 ^a^
F2	4.93 ± 1.46 ^a^	4.51 ± 0.22 ^a^	4.82 ± 0.21 ^a^	5.03 ± 0.19 ^a^	5.51 ± 0.17 ^a^
F3	4.73 ± 1.46 ^a^	4.81 ± 0.20 ^a^	4.55 ± 0.19 ^a^	4.70 ± 0.17 ^a^	5.40 ± 0.18 ^a^

Mean ± SD values (*n* = 55 well-trained panellists) with a similar superscript ^a^ in the same column were not significantly different (Kruskal–Wallis test, *p* > 0.05). C = control sample (without GABA); F1 = dark chocolate enriched with 0.05% GABA; F2 = 0.10% GABA; F3 = 0.15% GABA.

## Data Availability

Data is contained within the article.
